# *Toxocara* Seroprevalence among Clinically Healthy Individuals, Pregnant Women and Psychiatric Patients and Associated Risk Factors in Shandong Province, Eastern China

**DOI:** 10.1371/journal.pntd.0003082

**Published:** 2014-08-07

**Authors:** Wei Cong, Xiao-Xuan Zhang, Na Zhou, Chang-Zheng Yu, Jia Chen, Xiang-Yang Wang, Bing Li, Ai-Dong Qian, Xing-Quan Zhu

**Affiliations:** 1 State Key Laboratory of Veterinary Etiological Biology, Key Laboratory of Veterinary Parasitology of Gansu Province, Lanzhou Veterinary Research Institute, Chinese Academy of Agricultural Sciences, Lanzhou, Gansu Province, People′s Republic of China; 2 College of Animal Science and Technology, Jilin Agricultural University, Changchun, Jilin Province, People′s Republic of China; 3 Affiliated Hospital of Medical College, Qingdao University, Qingdao, Shandong Province, People′s Republic of China; 4 Weihai Wendeng Central Hospital, Weihai, Shandong Province, People′s Republic of China; 5 Wendeng Municipal Hospital, Weihai, Shandong Province, People′s Republic of China; 6 Wendeng People's Hospital, Weihai, Shandong Province, People′s Republic of China; National Institute of Parasitic Diseases, Chinese Center for Diseases Control and Prevention, China

## Abstract

**Background:**

Toxocarosis is a widespread zoonosis caused by the ascarid nematodes *Toxocara canis* and *Toxocara cati*, which primarily infect dogs and cats, respectively. Most human infections with *Toxocara* are asymptomatic; however, some infected individuals may develop a serious illness and even death. Nevertheless, epidemiological knowledge regarding the prevalence and risks associated with *Toxocara* infection is limited in China. Therefore, we performed a cross-sectional pilot study and estimated the seroprevalence of *Toxocara* infection in humans in Shandong Province, eastern China for the first time, from June 2011 to July 2013, involving clinically healthy individuals, pregnant women and psychiatric patients, aiming to attract public attention to *Toxocara* infection.

**Methodology/Principle Findings:**

Seroprevalence of *Toxocara* was determined using an enzyme-linked immunosorbent assay based on a cross-sectional study conducted in Qingdao and Weihai, Shandong Province, eastern China. Factors potentially associated with *Toxocara* infection were identified by logistic regression analysis. The overall *Toxocara* seroprevalence among the study population (n = 2866) was 12.25%, and a significantly higher seroprevalence in psychiatric patients (16.40%, 73/445) than that in clinically healthy individuals (13.07%, 187/1431) and pregnant women (9.19%, 91/990) was revealed. Univariate analyses suggested that keeping dogs at home (OR = 0.06, 95% CI 0.05–0.08, *P*<0.001), contact with cats and dogs (OR = 0.42, 95% CI 0.33–0.53, *P*<0.001) and exposure with soil (OR = 0.37, 95% CI 0.28–0.49, *P*<0.001) were risk factors associated with *Toxocara* infection.

**Conclusions/Significance:**

The present study revealed, for the first time, that human infection with *Toxocara* is common in eastern China, posing a significant public health concern. Increasing human and dog populations, population movements and climate change all will serve to increase the importance of this zoonosis. Further studies under controlled conditions are necessary to define potential morbidity associated with *Toxocara* infection.

## Author Summary

Toxocarosis, a typical neglected and underestimated human health problem, is caused by the ascarid nematodes *Toxocara canis* and *Toxocara cati*, which primarily infect dogs and cats, respectively. Previous studies have reported an increased risk for *Toxocara* infection in humans worldwide, especially in children and psychiatric patients. This pilot study was aimed to investigate the *Toxocara* serology in clinically healthy individuals, pregnant women and psychiatric patients in Shandong Province, eastern China using an enzyme-linked immunosorbent assay. The overall *Toxocara* seroprevalence among the study population (n = 2866) was 12.25%, and a significantly higher seroprevalence in psychiatric patients (16.40%, 73/445) than that in clinically healthy individuals (13.07%, 187/1431) and pregnant women (9.19%, 91/990) was revealed. Keeping dogs at home, contact with cats and dogs, and exposure with soil were found to be associated with *Toxocara* infection. Our findings indicate that human infection with *Toxocara* is common in eastern China, posing a significant public health concern.

## Introduction

Toxocarosis, a typical neglected and underestimated human health problem, is caused by the larval stages of *Toxocara canis*, the intestinal roundworms of dogs, and probably by the roundworm of cats (*Toxocara cati*) as well [1]–[3]. *T. canis*, the major cause of human toxocarosis, can reach a high prevalence owing to the large number of eggs excreted and the resistance of eggs to environmental conditions [4]. Humans can be infected by the accidental ingestion of embryonated *Toxocara* spp. eggs presented in contaminated soil or food, or by the ingestion of encapsulated larvae contained in the tissues of paratenic hosts [2]. Most human infections with *Toxocara* are asymptomatic; however, infective *Toxocara* larvae may migrate into internal organs via the blood and can result in a number of clinical syndromes such as visceral larva migrans (VLM), ocular larva migrans (OLM), neurotoxocarosis (NT), covert toxocarosis (CT) and eosinophilic meningoencephalitis (EME) [2]–[8].

The diagnosis of human toxocarosis is usually based on serological examinations, in combination with clinical presentations and the results of blood examinations [4]. Enzyme-Linked Immunosorbent Assay (ELISA) and Western Blotting (WB) are currently the most sensitive and reliable tools for detecting antibodies and circulating antigens [9]. However, WB is more expensive and labour-intensive than ELISA; thus alternatively, detecting reactive IgG by utilizing recombinant *T. canis* antigens as well as IgE antibodies in ELISA may expediently acquire convincing results [8].

Stray and domestic dogs and cats, especially from low income rural population, play a critical role in the transmission of *Toxocara* spp. offering environmental contamination, which extends the spreading of the infection among the human populations [10]. The growing numbers of dogs (pet and stray dogs) have resulted in closer contact between these animals and humans, enhancing the level of exposure [4]. In China, with the accelerated process of urbanization and the improvement of living standard, the number of pets raised (both in urban and rural areas) is increasing rapidly, and a series of problems are gradually emerging due to lack of quarantine and vaccination, ineffective market administration, non-standard pet hospital and environment contamination [11]. With respect to environmental characteristics, Qingdao and Weihai have a marine climate with moist air and abundant rainfall, which may contribute to become infective for the eggs [4]. Moreover, Qingdao and Weihai are important tourism and coastal open cities in eastern China, accepting a large number of tourists from home and abroad every year, and these mobility of human may introduce eggs of *Toxocara* spp. from other places. All of these may increase the risk of transmission of *Toxocara* spp. between humans and dogs and cats.

Considering that toxocarosis is one of the most common helminthosis worldwide, this neglected disease has been shown through seroprevalence studies to be especially prevalent in children from socio-economically disadvantaged populations both in the tropics and sub-tropical regions as well as in many developing countries [5], [10], [12]–[18]. However, epidemiological knowledge regarding the prevalence and risks associated with *Toxocara* infection is limited in China [19]. Therefore, we report the seroprevalence of *Toxocara* infection in humans in eastern China for the first time, including clinically healthy individuals, pregnant women and psychiatric patients, aiming to attract public attention to *Toxocara* infection.

## Methods

### Ethics statement

This study was approved before its commencement by the ethical committee of the Affiliated Hospital of Medical College, Qingdao University (Permit No. ECAHQU2011-006), Weihai Wendeng Central Hospital (Permit No. ECWCH2011-003), Wendeng Municipal Hospital (Permit No. ECWMH2011-008) and Wendeng People's Hospital (Permit No. ECWPH2011-002). The purpose and procedures of the study were explained to all participants, and a written informed consent was obtained from all of them. Parents/guardians provided informed consent on behalf of all child participants. The sera were collected with agreement from the volunteers or patients.

### Study design and study population

A cross-sectional study was conducted in Weihai and Qingdao, China. A total of 2866 study participants were recruited between June 2011 through July 2013, including 1431 clinically healthy individuals, 990 pregnant women and 445 psychiatric patients. People who participated in health screenings in the hospitals were considered as clinically healthy individuals. The pregnant women were recruited from women visited hospitals for antenatal follow-up or medication. Inclusion criteria for the pregnant women were: 1) pregnant women in any of the three trimesters of pregnancy; 2) aged 18 years and older; and 3) who were willing to participate in this study. The psychiatric patients were hospitalized for diagnosis or treatment. Inclusion criteria for the psychiatric patients were: 1) psychiatric inpatients; 2) aged 16 years and older; and 3) who accepted to participate in this study. The capacity to consent in psychiatric patients was determined through a clinical evaluation by hospital psychiatrists. Only psychiatric patients with capacity to consent and who accepted to participate were included in the study. In addition, a written informed consent was obtained from all participants and the next-of-kin of minor participants.

### Data collection

A structured questionnaire was used to assess risk factors, which included: study area, age, gender, ethnic groups, residential area, pregnancy status, stage of pregnancy, presence of cats and dogs at home, contact with cats and dogs, consumption of raw/undercooked meat, consumption of raw vegetables and fruits, source of drinking water and exposure to soil. In addition, Data of psychiatric patients were obtained from the patients, medical examination records, and informants. Classification of mental illnesses was performed according to the ICD-10 criteria [20].

### Sample collection and laboratory tests

Approximately 5 mL of venous blood samples were drawn from the participants in this study. Blood samples were left overnight at room temperature to allow clotting and centrifuged at 3000 rpm for 10 min. The sera were collected in Eppendorf tubes and stored at 4°C for 24–72 h until transported in an ice box to State Key Laboratory of Veterinary Etiological Biology, Key Laboratory of Veterinary Parasitology of Gansu Province, Lanzhou Veterinary Research Institute, Chinese Academy of Agricultural Sciences, Lanzhou, Gansu Province where they were kept at −20°C until tested. Serum samples were detected for anti-*Toxocara* IgG antibodies using a commercially available enzyme immunoassay “*Toxocara*” kit (Diagnostic Automation, Inc. Calabasas, CA, USA). Absorbance reading equal to or greater than 0.3 OD units was considered to be positive. All tests were performed following the instructions of the manufacturer [5].

### Statistical analysis

The strength of association between dependent (IgG seropositivity to *Toxocara*; yes/no) and independent variables 1) gender; 2) ethnic groups; 3) residence area; 4) keeping cats at home; 5) keeping dogs at home; 6) contact with cats and dogs; 7) raw vegetable consumption; 8) raw meat consumption; 9) exposure with soil; 10) source of water; was inferred by univariate logistic regression analysis using the SPSS 19.0 software package. Both dependent and independent variables were dichotomous variables. Odds ratio (OR) values were considered statistically significant if the 95% CI did not include 1. Probability (*P*) value<0.05 was considered as statistically significant in all the analyses.

## Results

### Seroprevalence among clinically healthy individuals

The clinically healthy individuals were randomly recruited from people who participated in health screenings in the hospitals. Table 1 shows the age, gender, ethnic groups, residence place and residence area distribution of clinically healthy individuals. A total of 1431 clinically healthy individuals of aged 15 to 93 years (mean 32.14 years) participated in this study. The majority (71.9%) of the clinically healthy individuals were in the age range of 20–39 years. In addition, the majority (95.6%) of their race were ethnic Han and most (70.9%) of the clinically healthy individuals lived in Weihai.

**Table 1:** Socio-demographic characteristics and seroprevalence of *Toxocara* infection among clinically healthy individuals in Shandong Province, eastern China.

Characteristic	No. subjects tested	Prevalence (%)(95%CI)	OR (95%CI)	*P* value
Age groups				
19 or less	133	15.04 (8.96–21.11)	Reference	
20–29	585	11.45 (8.87–14.03)	0.731 (0.426–1.253)	0.2528
30–39	444	14.19 (10.94–17.44)	0.934 (0.542–1.611)	0.8068
40–49	181	10.50 (6.03–14.96)	0.663 (0.338–1.298)	0.2281
50–59	45	17.78 (6.61–28.95)	1.222 (0.497–3.005)	0.6625
≥60	43	23.26 (10.63–35.88)	1.712 (0.730–4.016)	0.2128
Gender				
Male	475	7.16 (4.84–9.48)	Reference	
Female	956	16.00 (13.68–18.33)	2.471 (1.674–3.648)	<0.0001
Ethnic groups				
Ethnic Han	1368	13.23 (11.44–15.03)	Reference	
Ethnic Korean	63	9.52 (2.28–16.77)	0.690 (0.293–1.624)	0.3933
Residence place				
Qingdao	416	12.50 (9.32–15.68)	Reference	
Weihai	1015	13.30 (11.21–15.39)	1.074 (0.762–1.512)	0.6833
Residence area				
Urban	707	14.29 (11.71–16.87)	Reference	
Suburban or rural	724	11.88 (9.52–14.24)	0.809 (0.594–1.101)	0.1768

Anti-*Toxocara* IgG antibodies were detected in 187 (13.07%, 95% CI 11.32–14.81) of the 1431 clinically healthy individuals. The highest seroprevalence of *Toxocara* infection was detected in clinically healthy individuals aged ≥60 years old (23.26%, 95%CI 10.63–35.88) (Table 1). Moreover, statistically significant association between *Toxocara* seropositivity and gender was revealed in clinically healthy individuals (male: 7.16% *vs* female: 16.00%, OR = 2.471, 95% CI 1.674–3.648, *P*<0.0001).

### Seroprevalence among pregnant women

A total of 990 pregnant women who visited hospitals for antenatal follow-up or medication in Qingdao (n = 445) and Weihai (n = 545) were examined for anti-*Toxocara* IgG antibodies. Their age, residence place, residence area and trimester of pregnancy are shown in Table 2. The mean age of the 990 pregnant women participating in the study was 28.39 years (range 18–43). Over half (52.2%) of the pregnant women were in the age range of 26–30 years. The 52.8% of the pregnant women were in their first trimester of pregnancy. In addition, nearly half of the pregnant women lived in suburban or rural areas.

**Table 2:** Socio-demographic characteristics and seroprevalence of *Toxocara* infection among pregnant women in Shandong Province, eastern China.

Characteristic	No. subjects tested	Prevalence (%)(95%CI)	OR (95%CI)	*P* value
Age groups (years)				
25 or less	210	9.52 (5.55–13.49)	Reference	
26–30	517	8.51 (6.11–10.92)	0.884 (0.507–1.539)	0.6621
31–35	218	11.01 (6.85–15.16)	1.175 (0.628–2.198)	0.6130
>35	45	6.67 (0.00–13.96)	0.679 (0.193–2.389)	0.5437
Residence place				
Qingdao	445	9.89 (7.11–12.66)	Reference	
Weihai	545	8.62 (6.27–10.98)	0.860 (0.559–1.324)	0.4936
Residence area				
Urban	545	8.44 (6.11–10.77)	Reference	
Suburban or rural	445	10.11 (7.31–12.91)	1.220 (0.793–1.879)	0.3650
Trimester of pregnancy				
1^st^ trimester	523	9.94 (7.38–12.51)	Reference	
2^nd^ trimester	220	7.27 (3.84–10.70)	0.710 (0.396–1.274)	0.2492
3^rd^ trimester	247	9.31 (5.69–12.94)	0.930 (0.555–1.558)	0.7829

The overall seroprevalence of *Toxocara* infection among the pregnant women was 9.19% (91/990, 95% CI 7.39–10.99). Of these, 44 (9.89%) of 445 in Qingdao and 47 (8.62%) of 545 in Weihai were tested positive, but the difference was not statistically significant (OR = 0.860, 95% CI 0.559–1.324, *P*>0.05) (Table 2). The seroprevalence of *Toxocara* infection among the pregnant women who lived in suburban or rural areas (10.11%, 95% CI 7.31–12.91) was higher than that among those who lived in urban areas (8.44%, 95% CI 6.11–10.77) (OR = 1.220, 95% CI 0.793–1.879).

### Seroprevalence among psychiatric patients

The age, gender, ethnic groups and residence area distribution of the psychiatric patients are shown in Table 3. The mean age of the 445 psychiatric patients participating in the study was 37.18 years (range 16–91). Over half (54.2%) of the psychiatric patients were in the age range of 20–39 years. The 46.5% of the population in the psychiatric patients were males (n = 207), whereas 53.5% were females (n = 238). Moreover, the majority (85.8%) of their race were ethnic Han and most (71.2%) of the psychiatric patients lived in urban areas.

**Table 3:** Socio-demographic characteristics and seroprevalence of *Toxocara* infection among psychiatric patients in Shandong Province, eastern China.

Characteristic	No. subjects tested	Prevalence (%)(95%CI)	OR (95%CI)	*P* value
Age groups				
19 or less	44	18.18 (6.79–29.58)	Reference	
20–29	128	20.31 (13.34–27.28)	1.147 (0.476–2.762)	0.7595
30–39	113	12.39 (6.32–18.46)	0.636 (0.246–1.643)	0.3477
40–49	80	10.00 (3.43–16.57)	0.500 (0.173–1.441)	0.1935
50–59	40	20.00 (7.60–32.40)	1.125 (0.378–3.345)	0.8322
≥60	40	22.59 (9.56–35.44)	1.306 (0.450–3.796)	0.6227
Gender				
Male	207	20.29 (14.81–25.77)	Reference	
Female	238	13.03 (8.75–17.30)	0.558 (0.354–0.977)	0.0390
Ethnic groups				
Ethnic Han	382	16.75 (13.01–20.50)	Reference	
Ethnic Korean	63	14.29 (5.45–22.98)	0.828 (0.389–1.762)	0.6240
Residence area				
Urban	317	14.20 (10.35–18.04)	Reference	
Suburban or rural	128	21.88 (14.71–29.04)	1.692 (1.002–2.860)	0.0477

Table 4 shows clinical diagnosis data of the psychiatric patients based on their psychiatric disorders, which was performed according to the ICD-10 criteria. Patients suffered from organic, including symptomatic, mental disorders (F00–09) (n = 18), mental and behavioral disorders due to psychoactive substance use (F10–19) (n = 79), schizophrenia, schizotypal and delusional disorders (F20–29) (n = 34), mood (affective) disorders (F30–39) (n = 127), neurotic, stress-related and somatoform disorders (F40–49) (n = 65), mental retardation (F70–79) (n = 40), Alzheimer's disease (G30) (n = 33) and epilepsy (G40) (n = 49).

**Table 4:** Clinical diagnosis and seroprevalence of anti-*Toxocara* IgG antibodies in psychiatric patients in Shandong Province, eastern China.

Clinical Diagnosis	ICD-10 diagnosis	Patients with anti-*Toxocara* IgG antibodies			
		No. tested	No.	% (95%CI)	*P* value*
Epilepsy	G40	49	17	34.69 (21.37–48.02)	<0.0001
Affective disorder	F30, F39	13	1	7.69 (0.00–22.18)	0.5664
Somatoform disorder	F45	36	5	13.89 (2.59–25.19)	0.8853
Schizophrenia	F20	34	5	14.71 (2.80–26.61)	0.7797
Mental and behavioural disorders due to use of alcohol	F10, F18	53	8	15.09 (5.46–24.73)	0.6680
Mental and behavioural disorders due to use of drug	F13, F15, F19	26	2	7.69 (0.00–17.94)	0.4188
Obsessive-compulsive disorder	F42	29	5	17.24 (3.49–30.99)	0.5103
Mild depression	F32.051	23	3	13.03 (0.00–26.81)	0.9973
Moderate depression	F32.151	52	6	11.54 (2.86–20.22)	0.7475
Major depressive disorder	F32.251	39	3	7.69 (0.00–16.06)	0.3235
Mental retardation	F71–37, F78	40	7	17.50 (5.73–29.28)	0.4139
Alzheimer's disease	G30	33	7	21.21 (7.26–35.16)	0.1725
Dementia in Alzheimer's disease with early onset	F00.0	18	4	22.22 (3.02–41.43)	0.2539
Total		445	73	16.40 (12.96–19.85)	0.0752

*As compared with 13.07% seroprevalence of anti-*Toxocara* IgG antibodies in clinically healthy individuals (187/1431).

Seventy-three (16.40%) of the 445 psychiatric patients were positive for anti-*Toxocara* IgG antibodies indicating latent infection with *Toxocara* in psychiatric patients. Table 3 shows the seroprevalences of latent *Toxocara* infection in the populations studied according to their socio-demographic characteristics. Table 4 shows the seroprevalences of *Toxocara* infection in the patients according to their psychiatric disorders. The highest prevalence of latent *Toxocara* infection in psychiatric patients was found in patients aged ≥60 years old (22.59%, 95% CI 9.56–35.44) (Table 3). The seroprevalence in male psychiatric patients (20.29%, 95% CI 14.81–25.77) was significantly higher than that in female psychiatric patients (13.03%, 95% CI 8.75–17.30) (OR = 0.558, 95% CI 0.354–0.977, *P* = 0.0390). In addition, the seroprevalence of latent *Toxocara* infection in psychiatric patients who lived in suburban or rural areas (21.88%, 95% CI 14.71–29.04) was significantly higher than that in urban psychiatric patients (14.20%, 95% CI 10.35–18.04) (OR = 1.692, 95% CI 1.002–2.860, *P* = 0.0477). With respect to clinical data, statistically significant association between *Toxocara* seropositivity and psychiatric diagnosis was found. The seroprevalence of latent *Toxocara* infection in patients with epilepsy (17/49, 34.69%, 95%CI 21.37–48.02) was significantly higher than that in clinically healthy individuals (Table 4) (*P*<0.0001).

### Univariate logistic regression analysis

Comparing the total seropositive (351 subjects) with seronegative populations (2515 subjects), the univariate logistic regression analysis confirmed that keeping dogs at home (OR = 0.06, 95% CI 0.05–0.08, *P*<0.001), contact with cats and dogs (OR = 0.42, 95% CI 0.33–0.53, *P*<0.001) and exposure with soil (OR = 0.37, 95% CI 0.28–0.49, *P*<0.001) were risk factors associated with *Toxocara* infection. Other details are shown in Table 5.

**Table 5:** Odds ratio of the risk factors associated with seropositivity to *Toxocara* in the study population in Shandong province, eastern China.

Characteristic	No. subjects tested	Prevalence (%)(95%CI)	OR (95%CI)	*P* value
Age groups				
19 or less	189	15.87 (10.66–21.08)	Reference	
20–29	1331	11.20 (9.50–12.89)	0.668 (0.436–1.023)	0.0619
30–39	909	12.10 (9.98–14.22)	0.730 (0.471–1.131)	0.1572
40–49	269	10.04 (6.45–13.63)	0.591 (0.339–1.032)	0.0625
50–59	85	18.82 (10.51–27.13)	1.229 (0.629–2.400)	0.5455
≥60	83	22.89 (13.85–31.93)	1.573 (0.827–2.995)	0.1655
Gender				
Male	682	11.14 (8.78–13.51)	Reference	
Female	2184	12.59 (11.20–13.98)	1.15 (0.88–1.51)	0.3140
Ethnic groups				
Ethnic Han	2740	12.15 (10.93–13.38)	Reference	
Ethnic Korean	126	11.91 (6.25–17.56)	0.98 (0.56–1.70)	0.9334
Residence place				
Qingdao	861	11.15 (9.05–13.25)	Reference	
Weihai	2005	12.72 (11.26–14.18)	1.16 (0.91–1.49)	0.2403
Residence area				
Urban	1569	12.24 (10.62–13.86)	Reference	
Suburban or rural	1297	12.26 (10.47–14.04)	1.00 (0.80–1.25)	0.9858
Cats at home				
Yes	256	13.28 (9.12–17.44)	Reference	
No	2610	12.15 (10.89–13.40)	0.90 (0.62–1.32)	0.5969
Dogs at home				
Yes	288	56.94 (51.22–62.66)	Reference	
No	2578	7.25 (6.25–8.26)	0.06 (0.05–0.08)	<0.001
Contact with cats and dogs				
Yes	1312	17.23 (15.18–19.27)	Reference	
No	1554	8.04 (6.69–9.40)	0.42 (0.33–0.53)	<0.001
Raw vegetable consumption				
Yes	1915	12.59 (11.10–14.07)	Reference	
No	951	11.57 (9.53–13.60)	0.91 (0.71–1.16)	0.4337
Raw meat consumption				
Yes	1992	12.60 (11.14–14.06)	Reference	
No	874	11.44 (9.33–13.55)	0.90 (0.70–1.15)	0.3837
Exposure with soil				
Yes	1888	15.36 (13.73–16.99)	Reference	
No	978	6.24 (4.72–7.75)	0.37 (0.28–0.49)	<0.001
Source of water				
Tap	2019	12.28 (10.85–13.72)	Reference	
Well+river	847	12.16 (9.96–14.36)	0.99 (0.77–1.26)	0.9271

## Discussion

This pilot study investigated *Toxocara* seroprevalence in clinically healthy individuals, pregnant women and psychiatric patients in Shandong Province, eastern China, and potential factors associated with *Toxocara* infection were evaluated as well. The overall *Toxocara* IgG prevalence among the study population was 12.25%, which is slightly higher than that of a previous study conducted in Chengdu, China, where a 11.49% seroprevalence of *Toxocara* spp. infection was reported in children [19]. Such differences may due to several factors, such as geographical conditions, the type and size of population evaluated, life style of the population evaluated, as well as the specificity and sensitivity of the detection methods used. In the present study, a significantly higher seroprevalence of *Toxocara* seropositivity was found in psychiatric patients (16.40%) than in clinically healthy individuals (13.07%) and pregnant women (9.19%). Furthermore, seroprevalence increased along with age but not associated with gender, ethnic groups, or residence area. To the best of our knowledge, the past epidemiological knowledge regarding the prevalence and risk associated with *Toxocara* infection is limited in China [19], and this is the first report of *Toxocara* infection among clinically healthy adults, pregnant women and psychiatric patients in China.

In the present study, a significantly higher seroprevalence of *Toxocara* seropositivity in psychiatric patients than in clinically healthy individuals and pregnant women was found. Studies of toxocarosis in psychiatric inpatients have been conducted in Mexico [5] and Italy [21]. Similarly, the highest *Toxocara* seroprevalence found in the psychiatric patients in our study agrees with that found in a case-control seroprevalence study in Durango, Mexico [5], where researchers found a significantly higher seroprevalence of *Toxocara* seropositivity in psychiatric inpatients (4.7%) as compared with control subjects (1.1%). It is not clear why psychiatric patients had an increased *Toxocara* seroprevalence. However, during the investigation, we found that psychiatric patients were often in poor hygiene practices and many patients have had contacted with cats and dogs, and these risk factors might have contributed to an increased *Toxocara* exposure.

Several studies have demonstrated that contact with dogs is an important risk factor for toxocarosis [10], [22], [23]. Humans may become infected with *T. canis* via direct contact with dogs. Although stray dogs had a significantly higher prevalence of *T. canis* than domestic dogs, domestic dogs are the biggest risk for human exposure due to frequent contact with their owners [23]. *T. canis* eggs have been found in both the faeces and on the furs of domestic dogs in previous studies [23], [24]. Once the conditions become suitable, *T. canis* eggs may become embryonated in furs of domestic dogs. Therefore, direct contact with these dogs, such as petting, may increase the risk of transmission of *Toxocara* spp. [23], [24]. Our results revealed that the presence of dogs at home and contact with dogs and cats are risk factors for *Toxocara* infection in this study population. In addition, we found that female had higher seroprevalence than that in male in the present study, indicating that females are at higher risk of *Toxocara* infection in eastern China. This may be related to many factors. For example, females usually take more care of pet animals including dogs and cats at home, and moreover, females handle raw meat and vegetables more frequently than males because they spend more time on cooking at home. Thus it is more important to propagandize the knowledge of disease prevention in public, especially for the female groups.

Moreover, our data show that *Toxocara* exposure is the most frequent in the aged people of more than 60 years old. Although it is different from other studies [13], [14], it may be ascribed to some typical factors in China. First, the differences in seroprevalence may due to differences in the diagnostic methods, ecological and geographical factors. In addition, life style, dietary habit, number of dogs and cats and number of sample size among the study population may contribute to the differences in *Toxocara* seropositivity. More importantly, with the significant socio-economic advances and an increase in living standard, pet dogs and cats are very common in Chinese family, especially in the aged, leading to frequent contact with these reservoir hosts of *Toxocara* and thus the increased risks [3]. Therefore, our data and other studies have emphasized again that the presence of dogs in the household and contact with dogs are considered to be the major risk factor for the presence of *Toxocara* seropositivity in people [25], [26], proposing that the importance of toxocarosis is likely to grow due to the rapid growth of the human and dog populations and their increasing densities in urban areas without specific control initiatives.

Many studies have evaluated the consumption of raw or undercooked viscera or meat as a potential risk factor for *Toxocara* infection, and have shown their associations with human *Toxocara* seroprevalence [27]–[29]. However, in the present study no association between *Toxocara* seropositivity and the consumption of raw or undercooked viscera or meat was found, which may be related to the type and size of population evaluated. So, the association between toxocarosis and consumption of raw or undercooked viscera or meat warrants further research.

High human *Toxocara* seroprevalence has been reported in areas with documented soil contamination, and the risk for transmission may be increased in proportion to the degree of environmental contamination [30], [31]. Similarly, the present study reported that there was no significant difference in the *Toxocara* seroprevalence between urban and suburban or rural areas (*P*>0.05), but humans exposed with soil was significantly relevant to positive serology (*P*<0.001).

In conclusion, the present study revealed for the first time that human infection with *Toxocara* is common in eastern China, posing a significant public health concern. Increasing human and dog populations, population movements and climate change all will serve to increase the importance of this zoonosis [26]. Further studies under controlled conditions are necessary to further define potential morbidity associated with *Toxocara* infection. In addition, prevention efforts such as hand washing before eating and after soil contact, prevention of soil contamination in public areas by dog and cat feces, and preventive anthelmintic treatment of dogs and cats, starting at an early age, can be beneficial to minimize exposure to *Toxocara* spp. and help control potential morbidity associated with *Toxocara* infection.

## Supporting Information

Checklist S1 **STROBE checklist.**
(DOC)
