# Supplementary material for: Toxocara Seroprevalence among Clinically Healthy Individuals, Pregnant Women and Psychiatric Patients and Associated Risk Factors in Shandong Province, Eastern China
Source: PLoS Negl Trop Dis. 2014 Aug 7;8(8):e3082. doi: 10.1371/journal.pntd.0003082 (PMC4125144; doi:10.1371/journal.pntd.0003082)
Supplement: Checklist S1 — STROBE checklist. (DOC) [file pntd.0003082.s001.doc]

STROBE Statement—***cross-sectional studies***

|  | Item No |  |
| --- | --- | --- |
| **Title and abstract** | 1 | *Toxocara* Seroprevalence among Clinically Healthy Individuals, Pregnant Women and Psychiatric Patients and Associated Risk Factors in Shandong Province, Eastern China |
|  |  | (*b*) **Background:** Toxocarosis, a typical neglected and underestimated human health problem, most human infections with *Toxocara* are asymptomatic, however, some infected individuals may develop a serious illness and even death. Nevertheless, epidemiological knowledge regarding the prevalence and risks associated with *Toxocara* infection is limited in China.  **Objective:** To estimate the seroprevalence and associated risk factors of *Toxocara* infection in humans in Shandong Province, eastern China, involving clinically healthy individuals, pregnant women and psychiatric patients, aiming to attract public attention to *Toxocara* infection.  **Design:** Population-based cross-sectional study was conducted in Weihai and Qingdao, Shandong province, eastern China.  **Study population:** A total of 2866 study participants were recruited between June 2011 and July 2013, including 1431 clinically healthy individuals, 990 pregnant women and 445 psychiatric patients.  **Methodology:** Seroprevalance of *Toxocara* was determined using an enzyme-linked immunosorbent assay and factors potentially associated with *Toxocara* infection were identified by logistic regression analysis.  **Results:** The overall *Toxocara* seroprevalence among the study population (n=2866) was 12.25%, and a significantly higher seroprevalence in psychiatric patients (16.40%, 73/445) than that in clinically healthy individuals (13.07%, 187/1431) and pregnant women (9.19%, 91/990) was revealed. Univariate analyses suggested that keeping dogs at home (OR=0.06, 95% CI 0.05-0.08, *P*<0.001), contact with cats and dogs (OR=0.42, 95% CI 0.33-0.53, *P*<0.001) and exposure with soil (OR=0.37, 95% CI 0.28-0.49, *P*<0.001) were risk factors associated with *Toxocara* infection.  **Conclusions:** The present study revealed, for the first time, that human infection with *Toxocara* is common in eastern China, posing a significant public health concern. Increasing human and dog populations, population movements and climate change all will serve to increase the importance of this zoonosis. Further studies under controlled conditions are necessary to define potential morbidity associated with *Toxocara* infection. |
| Introduction | | |
| Background/rationale | 2 | Toxocarosis, a typical neglected and underestimated human health problem, is caused by the larval stages of *Toxocara canis*, the intestinal roundworms of dogs, and probably by the roundworm of cats (*Toxocara cati*) as well. Most human infections with *Toxocara* are asymptomatic, however, some infected individuals may develop a serious illness and even death. Previous studies have reported an increased risk for *Toxocara* infection in humans worldwide, especially in children and psychiatric patients. However, epidemiological knowledge regarding the prevalence and risks associated with *Toxocara* infection is limited in China. |
| Objectives | 3 | Our primary objectives were to 1) determine the seroprevalence of *Toxocara* infection in humans in eastern China, including clinically healthy individuals, pregnant women and psychiatric patients and 2) identify the risk factors associated with *Toxocara* infection in the study population. |
| Methods | | |
| Study design | 4 | We conducted a cross-sectional study to estimate the seroprevalence and associated risk factors of *Toxocara* infection in humans in Qingdao and Weihai, Shangdong Province, Eastern China. A total of 2866 study participants were recruited between June 2011 and July 2013, including 1431 clinically healthy individuals, 990 pregnant women and 445 psychiatric patients. |
| Setting | 5 | The cross-sectional study recruited participants from Affilliated Hospital of Medical College, Qingdao University, Weihai Wendeng Central Hospital, Wendeng Municipal Hospital, and Wendeng People’s Hospital between June 2011 and July 2013. People who participated in health screenings in the hospitals were considered as clinically healthy individuals. The pregnant women were recruited from women visited hospitals for antenatal follow-up or medication. The psychiatric patients were hospitalized for diagnosis or treatment. |
| Participants | 6 | People who participated in health screenings in the hospitals were considered as clinically healthy individuals. The pregnant women were recruited from women visited hospitals for antenatal follow-up or medication. Inclusion criteria for the pregnant women were: 1) pregnant women in any of the three trimesters of pregnancy; 2) aged 18 years and older; and 3) who were willing to participate in this study. The psychiatric patients were hospitalized for diagnosis or treatment. Inclusion criteria for the psychiatric patients were: 1) psychiatric inpatients; 2) aged 16 years and older; and 3) who accepted to participate in this study. The capacity to consent in psychiatric patients was determined through a clinical evaluation by hospital psychiatrists. Only psychiatric patients with capacity to consent and who accepted to participate were included in the study. In addition, a written informed consent was obtained from all participants and the next-of-kin of minor participants. |
| Variables | 7 | Serum samples from all participants were detected for anti-*Toxocara* IgG antibodies using a commercially available enzyme immunoassay ‘‘*Toxocara*’’ kit (Diagnostic Automation, Inc. Calabasas, CA, USA). Absorbance reading equal to or greater than 0.3 OD units was considered to be positive. The strength of association between dependent (IgG seropositivity to *Toxocara*; yes/no) and independent varibales 1) gender; 2) ethnic groups; 3) residence area; 4) keeping cats at home; 5) keeping dogs at home; 6) contact with cats and dogs; 7) raw vegetable consumption; 8) raw meat consumption; 9) exposure with soil; 10) source of water was inferred by univariate logistic regression analysis using the SPSS 19.0 software package. Both dependent and independent variables were dichotomous variables. Odds ratio (OR) values were considered statistically significant if the 95% CI did not include 1. Probability (*P*) value < 0.05 was considered as statistically significant in all the analyses. |
| Data sources/ measurement | 8* | Clinical diagnosis of the study population, including clinically healthy individuals, pregnant women and psychiatric patients, were obtained from the patients, medical examination records, and informants. Classification of mental illnesses was performed according to the ICD-10 criteria. In addition, a structured questionnaire was used to assess risk factors, which included: study area, age, gender, ethnic groups, residential area, pregnancy status, stage of pregnancy, presence of cats and dogs at home, contact with cats and dogs, consumption of raw/undercooked meat, consumption of raw vegetables and fruits, source of drinking water and exposure to soil. These variables were selected based on literature. |
| Bias | 9 | First, our study participants might not represent the general clinically healthy individuals, pregnant women and psychiatric patients population due to the potential limitation of enrollment methods. Therefore, potential selection bias should be considered when interpret our results. Second, serology could not clearly indicate the infection status as current infection or past infection, potential bias caused by such misclassification could not be eliminated. |
| Study size | 10 | The number of participants in these areas who awere willing to participate in this study during the study period determined the sample size. |
| Quantitative variables | 11 | People who participated in health screenings in the hospitals were considered as clinically healthy individuals. The pregnant women were recruited from women visited hospitals for antenatal follow-up or medication. The psychiatric patients were hospitalized for diagnosis or treatment. Univariate logistic regression analysis using the SPSS 19.0 software package was used to assess the risk factors of each groups, and at last the same analysis method was used the risk factors between the whole study population and *Toxocara* infection. |
| Statistical methods | 12 | (*a*) The strength of association between dependent (IgG seropositivity to *Toxocara*; yes/no) and independent varibales, was inferred by univariate logistic regression analysis using the SPSS 19.0 software package. Both dependent and independent variables were dichotomous variables. Odds ratio (OR) values were considered statistically significant if the 95% CI did not include 1. Probability (*P*) value < 0.05 was considered as statistically significant in all the analyses. |
|  |  | (*b*) Univariate logistic regression analysis using the SPSS 19.0 software package was used to infer the strength of association between dependent (IgG seropositivity to *Toxocara*; yes/no) and independent varibales, Probability (*P*) value < 0.05 was considered as statistically significant in all the analyses. Odds ratio (OR) values were considered statistically significant if the 95% CI did not include 1. |
|  |  | (*c*) Our missing data analysis procedures used missing completely at random (MCAR), and we directly deleted the case with missing data. |
|  |  | (*d*) None |
|  |  | (*e*) None |
| Results | | |
| Participants | 13* | (a) A total of 2866 study participants were recruited between June 2011 and July 2013, including 1431 clinically healthy individuals, 990 pregnant women and 445 psychiatric patients. Clinical diagnosis of the study population, including clinically healthy individuals, pregnant women and psychiatric patients, were obtained from the patients, medical examination records, and informants. Classification of mental illnesses was performed according to the ICD-10 criteria. |
|  |  | (b) The main reasons for non-participation were the participants were nonresponse, refusal and and other reasons (refusal by consultant or general practitioner, non-English and non-Chinese speaking). |
|  |  | 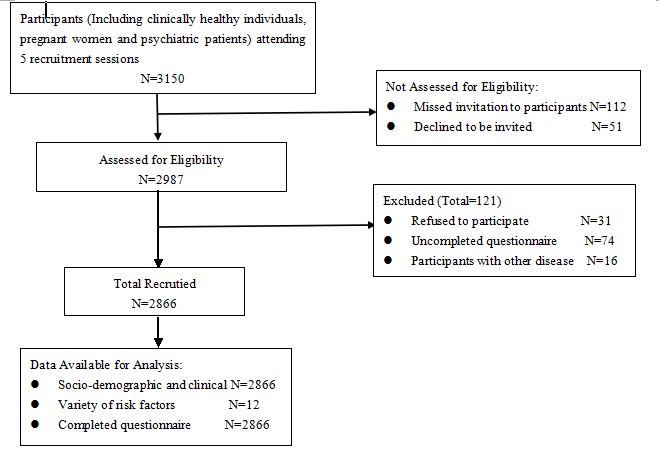(c) |
| Descriptive data | 14* | (a)   | **Table 1.** Characteristic of the study population in Shandong Province, eastern China | | | | | --- | --- | --- | --- | | Characteristic | No. subjects tested | | | | clinically healthy individuals | pregnant women | psychiatric patients | | Age groups |  |  |  | | 19 or less | 133 | 89 | 44 | | 20-29 | 585 | 587 | 128 | | 30-39 | 444 | 296 | 113 | | 40-49 | 181 | 20 | 80 | | 50-59 | 45 | 0 | 40 | | ≥60 | 43 | 0 | 40 | | Gender |  |  |  | | Male | 475 | 0 | 207 | | Female | 956 | 990 | 238 | | Ethnic groups |  |  |  | | Ethnic Han | 1368 | 990 | 382 | | Ethnic Korean | 63 | 0 | 63 | | Residence place |  |  |  | | Qingdao | 416 | 445 | 0 | | Weihai | 1015 | 545 | 445 | | Residence area |  |  |  | | Urban | 707 | 545 | 317 | | Suburban or rural | 728 | 445 | 128 |  | **Table 2**. Clinical diagnosis of psychiatric patients in Shandong Province, eastern China. | | | | --- | --- | --- | | **Clinical Diagnosis** | **ICD-10 diagnosis** | **No. tested** | | Epilepsy | G40 | 49 | | Affective disorder | F30, F39 | 13 | | Somatoform disorder | F45 | 36 | | Schizophrenia | F20 | 34 | | Mental and behavioural disorders due to use of alcohol | F10, F18 | 53 | | Mental and behavioural disorders due to use of drug | F13, F15, F19 | 26 | | Obsessive-compulsive disorder | F42 | 29 | | Mild depression | F32.051 | 23 | | Moderate depression | F32.151 | 52 | | Major depressive disorder | F32.251 | 39 | | Mental retardation | F71-37, F78 | 40 | | Alzheimer's disease | G30 | 33 | | Dementia in Alzheimer's disease with early onset | F00.0 | 18 | | Total |  | 445 | |
|  |  | (b)   \| **Table.** The number of participants with missing data of each groups \| \| \| \| \| --- \| --- \| --- \| --- \| \|  \| clinically healthy individuals \| pregnant women \| psychiatric patients \| \| Refused to participate \| 5 \| 12 \| 14 \| \| Uncompleted questionnaire \| 58 \| 8 \| 7 \| \| Suffering from other diseases \| 16 \| 0 \| 0 \| \| Total \| 80 \| 20 \| 21 \| |
| Outcome data | 15* | | **Table.** Seroprevalence of *Toxocara* infection in the study population. | | | | | --- | --- | --- | --- | | Groups | No.tested | No.positive | Prevalence (95%CI) | | clinically healthy individuals | 1431 | 187 | 13.07% (11.32-14.81) | | pregnant women | 990 | 91 | 9.19% (7.39-10.99) | | psychiatric patients | 445 | 73 | 16.40% (12.96-19.85) | | Total | 2866 | 351 | 12.25% (11.05-13.45) | |
| Main results | 16 | (*a*)   | **Table 1.** Seroprevalence of *Toxocara* infection in the study population. | | | | | --- | --- | --- | --- | | Groups | No.tested | No.positive | Prevalence (95%CI) | | clinically healthy individuals | 1431 | 187 | 13.07% (11.32-14.81) | | pregnant women | 990 | 91 | 9.19% (7.39-10.99) | | psychiatric patients | 445 | 73 | 16.40% (12.96-19.85) | | Total | 2866 | 351 | 12.25% (11.05-13.45) |  | **Table 2.**  Risk factors associated with seropositivity to *Toxocara* in the study population | | | | | | --- | --- | --- | --- | --- | | **Characteristic** | **No. tested** | **Prevalence (%)(95%CI)** | **OR**  **(95%CI)** | ***P* value** | | Age groups |  |  |  |  | | 19 or less | 189 | 15.87  (10.66-21.08) | Reference |  | | 20-29 | 1331 | 11.20  (9.50-12.89) | 0.668 (0.436-1.023) | 0.062 | | 30-39 | 909 | 12.10  (9.98-14.22) | 0.730  (0.471-1.131) | 0.157 | | 40-49 | 269 | 10.04  (6.45-13.63) | 0.591  (0.339-1.032) | 0.063 | | 50-59 | 85 | 18.82  (10.51-27.13) | 1.229  (0.629-2.400) | 0.546 | | ≥60 | 83 | 22.89  (13.85-31.93) | 1.573  (0.827-2.995) | 0.166 | | Gender |  |  |  |  | | Male | 682 | 11.14  (8.78-13.51) | Reference |  | | Female | 2184 | 12.59  (11.20-13.98) | 1.15  (0.88-1.51) | 0.314 | | Ethnic groups |  |  |  |  | | Ethnic Han | 2740 | 12.15  (10.93-13.38) | Reference |  | | Ethnic Korean | 126 | 11.91  (6.25-17.56) | 0.98  (0.56-1.70) | 0.933 | | Residence place |  |  |  |  | | Qingdao | 861 | 11.15  (9.05-13.25) | Reference |  | | Weihai | 2005 | 12.72  (11.26-14.18) | 1.16  (0.91-1.49) | 0.240 | | Residence area |  |  |  |  | | Urban | 1569 | 12.24  (10.62-13.86) | Reference |  | | Suburban or rural | 1297 | 12.26  (10.47-14.04) | 1.00  (0.80-1.25) | 0.986 | | Cats at home |  |  |  |  | | Yes | 256 | 13.28  (9.12-17.44) | Reference |  | | No | 2610 | 12.15  (10.89-13.40) | 0.90  (0.62-1.32) | 0.600 | | Dogs at home |  |  |  |  | | Yes | 288 | 56.94  (51.22-62.66) | Reference |  | | No | 2578 | 7.25  (6.25-8.26) | 0.06  (0.05-0.08) | <0.01 | | Contact with cats and dogs |  |  |  |  | | Yes | 1312 | 17.23  (15.18-19.27) | Reference |  | | No | 1554 | 8.04  (6.69-9.40) | 0.42  (0.33-0.53) | <0.01 | | Raw vegetable consumption |  |  |  |  | | Yes | 1915 | 12.59  (11.10-14.07) | Reference |  | | No | 951 | 11.57  (9.53-13.60) | 0.91  (0.71-1.16) | 0.434 | | Raw meat consumption |  |  |  |  | | Yes | 1992 | 12.60  (11.14-14.06) | Reference |  | | No | 874 | 11.44  (9.33-13.55) | 0.90  (0.70-1.15) | 0.384 | | Exposure with soil |  |  |  |  | | Yes | 1888 | 15.36  (13.73-16.99) | Reference |  | | No | 978 | 6.24  (4.72-7.75) | 0.37  (0.28-0.49) | <0.01 | | Source of water |  |  |  |  | | Tap | 2019 | 12.28  (10.85-13.72) | Reference |  | | Well + river | 847 | 12.16  (9.96-14.36) | 0.99  (0.77-1.26) | 0.927 | |
|  |  | (*b*) None |
|  |  | (*c*) None |
| Other analyses | 17 | None |
| Discussion | | |
| Key results | 18 | The overall *Toxocara* seroprevalence among the study population (n=2866) was 12.25%, and a significantly higher seroprevalence in psychiatric patients (16.40%, 73/445) than that in clinically healthy individuals (13.07%, 187/1431) and pregnant women (9.19%, 91/990) was revealed. Univariate analyses suggested that keeping dogs at home (OR=0.06, 95% CI 0.05-0.08, *P*<0.001), contact with cats and dogs (OR=0.42, 95% CI 0.33-0.53, *P*<0.001) and exposure with soil (OR=0.37, 95% CI 0.28-0.49, *P*<0.001) were risk factors associated with *Toxocara* infection.The present study revealed, for the first time, that human infection with *Toxocara* is common in eastern China, posing a significant public health concern. |
| Limitations | 19 | First, our study participants might not represent the general clinically healthy individuals, pregnant women and psychiatric patients population due to the potential limitation of enrollment methods. Therefore, potential selection bias should be considered when interpret our results. Second, serology could not clearly indicate the infection status as current infection or past infection, potential bias caused by such misclassification could not be eliminated. Moreover, molecular identification, taxonomy, genetic variation and diagnosis of *Toxocara* spp. should be considered in further studies. Third, cross-sectional study design has its limitation on association analysis. Therefore, our results need to be proved in further large-scale case-control studies or prospective studies. |
| Interpretation | 20 | The present study revealed for the first time that human infection with *Toxocara* is common in eastern China, posing a significant public health concern. Increasing human and dog populations, population movements and climate change all may serve to increase the importance of this zoonosis. Further studies under controlled conditions are necessary to define potential morbidity associated with *Toxocara* infection. |
| Generalisability | 21 | The present study was firstly revealed the seroprevalence and risk factors of *Toxocara* infection in clinically healthy individuals, pregnant women and psychiatric patients in Shandong Province, eastern China, which provided the base-line data for taking integrated strategies and measures for the effective prevention and control of *Toxocara* infection in humans and we believe that this information will be significant for researching the *Toxocara* infection in hunmans in China. |
| Other information | | |
| Funding | 22 | Project support was provided by the International Science & Technology Cooperation Program of China (Grant No. 2013DFA31840) and the Science Fund for Creative Research Groups of Gansu Province (Grant No. 1210RJIA006). |

*Give information separately for exposed and unexposed groups.

**Note:** An Explanation and Elaboration article discusses each checklist item and gives methodological background and published examples of transparent reporting. The STROBE checklist is best used in conjunction with this article (freely available on the Web sites of PLoS Medicine at http://www.plosmedicine.org/, Annals of Internal Medicine at http://www.annals.org/, and Epidemiology at http://www.epidem.com/). Information on the STROBE Initiative is available at www.strobe-statement.org.
